# ENOX2-based early detection (ONCOblot) of asbestos-induced malignant mesothelioma 4–10 years in advance of clinical symptoms

**DOI:** 10.1186/s12014-016-9103-3

**Published:** 2016-01-22

**Authors:** D. James Morré, Brandon Hostetler, David J. Taggart, Dorothy M. Morré, A. W. Musk, Bruce W. S. Robinson, Jenette Creaney

**Affiliations:** MorNuCo, Inc, Purdue University Research Park, 1201B Cumberland Avenue, West Lafayette, IN USA; National Centre for Asbestos Related Disease, University of Western Australia, Perth, Australia; School of Medicine and Pharmacology, University of Western Australia, Perth, Australia; Department of Respiratory Medicine, Sir Charles Gairdner Hospital, Nedlands, Australia; School of Population Health, University of Western Australia, Perth, Australia

**Keywords:** Malignant mesothelioma, ENOX2, Early detection, ONCOblot tissue of origin cancer test, Serum analysis, Asbestos

## Abstract

**Background:**

Malignant mesothelioma is an aggressive, almost uniformly fatal tumor, caused primarily by exposure to asbestos. In this study, serum presence of mesothelioma-specific protein transcript variants of ecto-nicotinamide adenine dinucleotide oxidase disulfide-thiol exchanger 2 (ENOX2), a recently identified marker of malignancy, were investigated using the ONCOblot tissue of origin cancer detection test.

**Methods:**

Sequential serum samples collected from asbestos-exposed individuals prior to the development of frank mesothelioma were assayed for ENOX2 presence by 2-D gel immunoblot analysis to determine how long in advance of clinical symptoms mesothelioma-specific ENOX2 transcript variants could be detected.

**Results:**

Two mesothelioma-specific ENOX2 protein transcript variants were detected in the serum of asbestos-exposed individuals 4–10 years prior to clinical diagnosis of malignant mesothelioma (average 6.2 years). Either one or both ENOX2 protein transcript variants indicative of malignant mesothelioma were absent in 14 of 15 subjects diagnosed with benign pleural plaques either with or without accompanying asbestosis.

**Conclusions:**

In a population of asbestos-exposed subjects who eventually developed malignant mesothelioma, ENOX2 protein transcript variants characteristic of malignant mesothelioma were present in serum 4–10 years in advance of clinical symptoms. As with all biomarker studies, these observations require validation in a larger, independent cohort of patients and should include prospective as well as retrospective sampling.

## Background

Malignant mesothelioma is an aggressive, almost uniformly fatal, asbestos-induced cancer [1, 2]. It is a tumor of the serosal cavities, predominantly of the pleura and is generally widespread throughout the cavity at the time of presentation. Patients who are treated with supportive care have a median survival of only 9 months [3]. Those treated with the best available chemotherapy (pemetrexed and cisplatinum) have an average increased survival of only 10 weeks [4], with a median survival between 9 and 14 months [5]. However, in select patients with early-stage epithelial disease who undergo extra pleural pneumonectomy, followed by adjunct chemotherapy and radiotherapy, 5-year survival rates of 46 % have been reported [6]. Therefore, like most cancers, early detection of malignant mesothelioma has the potential to improve patient outcomes [7].

Recently, the ONCOblot tissue of origin cancer detection test, a serum-based method for cancer detection, has been described [8]. The test is based on the discovery that there are cancer-specific transcript variants of ecto-nicotinamide adenine dinucleotide oxidase disulfide-thiol exchanger 2 (ENOX2) [8, 9] and consists of 2-D gel electrophoretic separation of serum proteins followed by immunoblot analysis with an ENOX2-specific recombinant antibody. ENOX2 belongs to a family of cell surface proteins that oxidize reduced pyridine nucleotides [NAD(P)H] and are essential for cell enlargement and growth [10]. At least 20 tissue of origin specific patterns of ENOX2 transcript variants have been described (and/or combinations of isoforms) indicative of the cancer tissue of origin [8]. These ENOX2 proteins are shed into the circulation and can be detected in some early stage cancers, including: breast, lung, colon, prostate and ovarian cancer (Table 1).

**Table 1:** Table of ranges

Cancer	**N**	Acceptable ranges					
		Protein 1		Protein 2		Protein 3	
		MW (kDa)	pI (pH)	MW (kDa)	pI (pH)	MW (kDa)	pI (pH)
Bladder	25	63–66	4.2–5.6	42–48	4.1–4.8		
Blood cell (total)	88	34–47	3.5–4.5				
Breast	538	64–69	4.2–4.9				
Cervical	37	90–100	4.2–5.4				
Colorectal	90	80–96	4.4–5.4	50–65	4.2–5.3	33–46	3.8–5.2
Endometrial (uterine)	60	67–71	4.2–5.1	41–48	3.7–5.4		
Esophageal	9	42–47	4.6–5.2				
Gastric	10	120–188	4.7–5.5	50–62	4.5–5.6	45–53	2.4–3.6
Hepatocellular	19	58–70	4.5–5.0	34–40	4.1–5.2		
Kidney (renal cell)	21	69–73	4.7–5.4	54–61	4.1–5.2	38–43	3.7–4.3
Leukemia^a^	32	34–45	3.5–4.5				
Lung (total)	103	52–56	4.1–5.3				
Lung, non-small cell^a^	71	54–56	4.7–5.3				
Lung, small-cell^a^	32	52–53	4.1–4.6				
Lymphoma^a^	33	43–45	3.5–4.5				
Melanoma	39	37–41	4.6–5.3				
Mesothelioma	25	60–68	3.8–4.1	38–44	3.8–4.6		
Myeloma^a^	23	38–47	3.6–4.5				
Ovarian	102	72–90	3.7–5.0	37–47	3.7–5.0		
Pancreatic	62	48–51	3.9–5.4				
Prostate	182	71–88	5.1–6.5				
Sarcoma	22	50–55	5.2–5.6	37–45	4.3–4.9		
Squamous cell	46	57–68	5.0–5.4				
Testicular germ cell	5	61–62	5.0–5.4	42–45	4.4–4.7		
Thyroid follicular	14	48–56	4.7–5.1	37–42	4.5–5.2		
Thyroid papillary	22	56–67	4.5–5.0	37–44	3.2–3.6		
Totals	1519						

^a^Bracketed entries are aggregate cancers or subsets already represented in the totals as aggregates (lung) or subsets (blood cell cancers)

ENOX2 transcript variants of specific molecular weights and isoelectric points (pIs) are produced uniquely by patients with cancer [10]. Identification of cancer presence by detecting ENOX2 transcript variants produces a low incidence of both false positives and false negatives (>1 % for subjects with clinically confirmed cancers), as they are molecular signature molecules produced specifically by cancer cells and are absent from non-cancer cells [10]. The predictive correlation between ONCOblot findings and the onset of cancer is based on findings that support ENOX2 as a marker of cancer presence [11].

Considerable interest exists in the development of early screening tests for malignant mesothelioma in asbestos-exposed populations. Therefore, the present study was undertaken to determine if cancer-specific ENOX2 transcript variants might serve as biomarkers to detect the presence of malignant mesothelioma in advance of clinical symptoms. To this end, serum samples, collected prior to diagnosis as part of a prospective cohort study [12, 13], were examined by using the ONCOblot test. This examination of serum samples from asbestos-exposed subjects revealed that patients with a clinically confirmed diagnosis of malignant mesothelioma produced a consistent pattern of two ENOX2 transcript variants. These two transcript variants were detected well in advance of clinical symptoms. These findings indicate that the ONCOblot Tissue of Origin Cancer Detection Test might, if validated in other studies, provide a useful addition to the diagnostic repertoire of tests for the early detection of malignant mesothelioma [14, 15].

## Results

### Study population characteristics

**Fig. 1:**
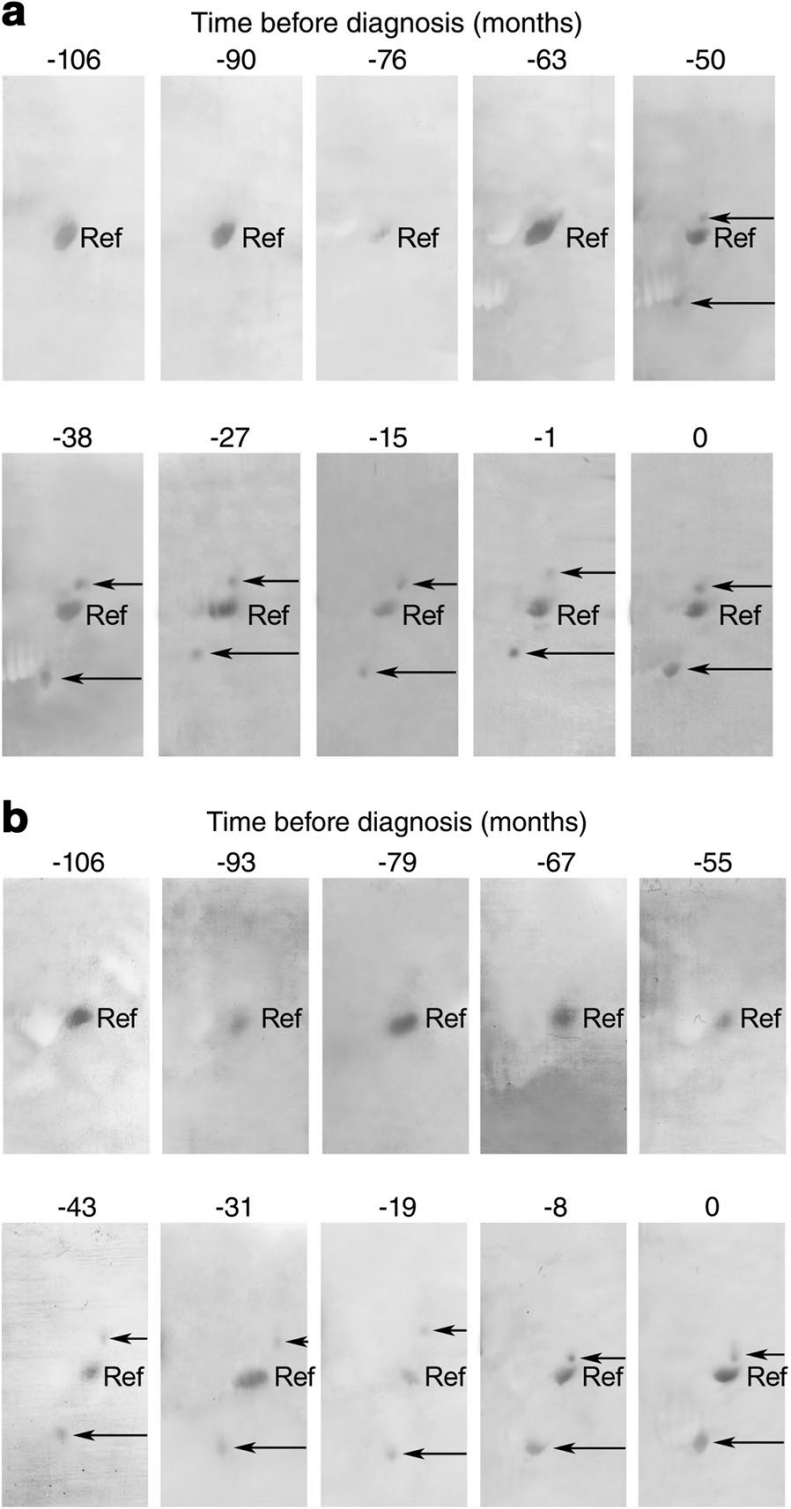
ONCOblot images. Images are from patient ID 2101 (**a**) and patient ID 2215 (**b**), beginning 106 months (−106) before diagnosis of asbestos-induced malignant mesothelioma. Isoelectric focusing was in the first dimension, pH range 3–5 shown, with sodium dodecyl sulfate gel electrophoresis in the second dimension with comparisons to a standard reference protein, α-fetuin. The α-fetuin reference protein is common to all non-cancer and cancer patient sera. The *long arrows* indicate the higher molecular weight transcript variant (Protein 1) and the *short arrows* indicate the lower molecular weight ENOX2 transcript variant (Protein 2)

**Fig. 2:**
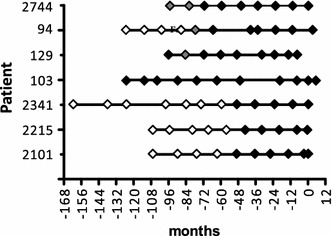
Summary diagram. Serial assays of the seven male subjects, median age of diagnosis 67 years, beginning 168–96 months before diagnosis of asbestos-induced malignant mesothelioma are represented. *Solid symbols—*both Protein 1 and Protein 2 evident. *Open symbols*—neither Protein 1 nor Protein 2 evident. *Shaded symbols*—only Protein 1 evident

Sera from 17 individuals with confirmed malignant mesothelioma were studied (Table 2). The majority of the malignant mesothelioma cases were of epithelial histology and all occurred in the pleural cavity. The majority of cases were male and the mean age of diagnosis was 67. The median survival for this group of patients was 24 months (95 % CI 20–30) after clinical diagnosis. For seven individuals, annual pre-diagnosis samples were available for analysis (Figs. 1a,b; 2). Sera from 15 asbestos-exposed subjects but free of malignancy were analyzed in parallel (Table 3). These subjects had a variety of benign lung and pleural disease and were of a similar age to the malignant mesothelioma patients at the time of serum collection (mean age 72 years). The asbestos-exposed control group included two females. For three individuals, annual serum samples were available for analysis (Table 3).

**Table 2:** Molecular weights and isoelectric points of mesothelioma-specific ENOX2 transcript variants from analyses of 17 confirmed male malignant mesothelioma patients

Patient	Age at sample	Protein 1		Protein 2	
		kDa	pH	kDa	pH
7457	70	60	3.8	38	4.4
7716	63	61	3.9	38	4.4
6500	53	60	3.8	38	4.4
2101	67	61	4.0	40	4.5
2215	75	61	3.9	42	3.8
94	66	63	3.8	42	3.8
103	54	68	3.9	41	4.1
129	64	66	3.8	39	4.3
2341	65	68	3.9	39	4.3
2744	68	63	4.0	41	3.9
7744	63	67	4.0	43	4.3
9484	73	65	3.8	42	4.6
9394	72	65	4.0	42	4.4
9111	72	67	3.8	42	4.2
9113	67	63	4.0	43	4.3
9446	63	62	3.9	44	4.5
9926	76	64	3.9	42	4.3
Mean		63.6	3.9	40.9	4.3
Standard deviation		±2.7	±0.1	±1.9	±0.2

**Table 3:** ENOX2 transcript variants and quantitation by spot size from analyses of 15 subjects (mean age 72) diagnosed with benign, asbestos-related lesions

Pt. ID	Gender	Diagnosis	Disease-free (years)	Protein 1 diam^a^ (mm)	Protein 2 diam^a^ (mm)
1268	M	Plaques + Asbestosis	1	2.0	1.3
			2	2.0	1.3
			4	1.5	0.8
			5	1.5	1.0
			7	1.0	1.3
			8	1.0	1.0
			10	0.8	1.3
1542	M	Plaques	1	NS	NS
			2	NS	1.6
			4	NS	1.8
			6	NS	1.6
			9	NS	1.3
			10	NS	1.3
			11	NS	1.6
1842	M	Plaques + Asbestosis	1	NS	NS
2374	M	Plaques + Asbestosis		NS	NS
2397	M	Plaques		NS	NS
2426	M	Plaques + Asbestosis		NS	NS
3720	M	Plaques		NS	NS
3768	M	Plaques		NS	NS
3846	M	Plaques		NS	NS
4288	F	Plaques + Asbestosis	8	2.2	NS
			9	2.0	NS
			10	1.8	NS
4334	M	Plaques + Asbestosis		NS	1.5
4476	M	Plaques		NS	1.4
9629	M	Plaques		NS	NS
9676	M	Plaques + Asbestosis		NS	NS
10011	F	Plaques		1.6	NS

^a^Spot diameter in mm

NS = no spot

### ENOX2 transcript variants indicative of malignant mesothelioma

The signature pattern of ENOX2 isoforms produced by malignant mesothelioma consisted of two ENOX2 transcript variants (Table 1; Fig. 1a, b). Consistently, two isoforms of ENOX2 were detected in sera samples collected from subjects an average 7.5 months (SD = 8) after confirmed diagnosis of malignant mesothelioma (Table 2). The larger ENOX2 transcript variant (Protein 1) had a molecular weight of 64 ± 2.7 kDa and a pI of pH 3.9 ± 0.1. The smaller variant (Protein 2) had a molecular weight of 41 ± 1.9 kDa and a pI of pH 4.3 ± 0.2. Both were detected in all 17 patients. The range in size and pI was 60–68 kDa, pH 3.8–4.1 for Protein 1 and 38–44 kDa, pH 3.8–4.6 for Protein 2 (Table 1).

### ENOX2 transcript variants detected in pre-diagnosis serum samples

For seven mesothelioma patients, annual serum samples were available before clinical diagnosis. For all seven, both ENOX2 protein isoforms were detected in pre-diagnostic serum samples (Figs. 1a, b; 2) available at least 4 years before diagnosis. For one subject, ENOX2 was detected 10 years prior to diagnosis (Fig. 2). While in five subjects both the Protein 1 and Protein 2 were detected at the same time point, expression of only Protein 1 was encountered in advance of Protein 2 in serial sample sets from Patients ID 94 and 2774 (Fig. 2). Also, for one subject (ID 129), Protein 2 was detected intermittently between 96 and 72 months prior to diagnosis. Overall, both of the mesothelioma-specific ENOX2 transcript variants were detected 4–10 years in advance of clinical symptoms and with an average of 6.2 ± 2.6 years in advance of clinical symptoms (Fig. 2).

### Subjects diagnosed with benign pleural plaques alone or with accompanying asbestosis

Of the fifteen asbestos-exposed subjects with benign pleural plaques either alone, or with accompanying asbestosis (Table 3), ENOX2 proteins were not detected in the sera of nine subjects (i.e., 60 %). Only one subject (ID 1268), was positive for both mesothelioma specific ENOX2 protein transcripts. Of the remainder, three subjects (ID 1542, 4334, and 4476) expressed only Protein 2 and two subjects (ID 4288 and 10011) expressed only Protein 1 (Table 3). Of the benign subjects exhibiting only Protein 1, both were female. For three subjects, longitudinal annual serum samples were available. For patient ID 1268, both ENOX2 protein isoforms were detected over a 10 year period (Table 3). This individual remains clinically malignancy-free 1.5 years after the last sample was analyzed. He has extensive pleural plaques, has never smoked and has a normal serum mesothelin level (data not shown). Only one of the ENOX2 isoforms was expressed in the other two benign patients examined longitudinally, though the protein was present over several years. Patient ID 4288 died approximately 1.5 years after the last sample was analyzed from non-malignant causes. Patient ID 1542 remains alive approximately a year after the last sample was examined.

### Correlation between clinical diagnosis and ENOX2 spot diameter

**Fig. 3:**
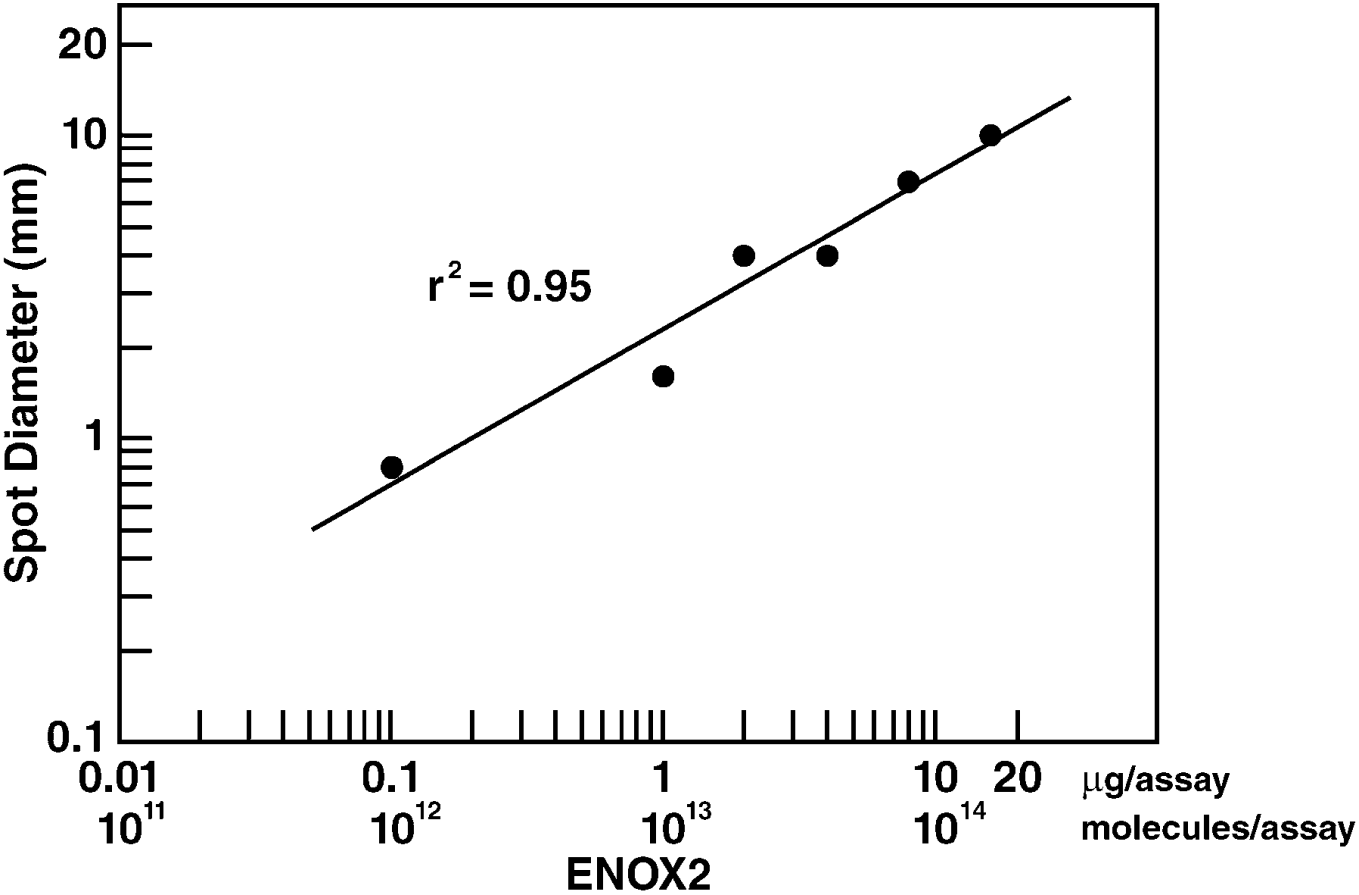
Log-log linear relationship between spot diameter and amount of ENOX2 protein. Varying amounts of purified, recombinant ENOX2 were analyzed by immunoblot. The log of the diameter of the detected ENOX2 spot was then plotted as a function of the log of the amount of ENOX2 loaded

For subjects who developed mesothelioma, the spot diameter at the earliest date of detection for both Protein 1 and Protein 2 was 1.95 ± 0.3 mm (not shown). The diameter remained more or less constant, increasing slightly to 2.3 ± 0.3 mm at the last date prior to mesothelioma diagnosis and a diameter of 3.2 ± 0.9 mm within the year following diagnosis. This represented approximately a twofold increase in serum ENOX2 concentration between the initial date of early detection and the clinical diagnosis of frank mesothelioma (Fig. 3).

In contrast, the amount of ENOX2 detected in the sera of time series subjects who have not developed mesothelioma (Table 3) either remained constant or declined. For subject ID 1268, spot diameter declined progressively from 2.0 to 0.8 mm between disease-free year 1 and 10. This decrease in spot diameter for Protein 1 for subject 1268 represents a reduction in the circulating ENOX2 concentration of approximately 90 % during the observation period, based on the relationship shown in Fig. 3. For subject ID 4288, expressing only Protein 1, the spot diameter declined from 2.2 to 1.8 mm between disease-free year 8 and 10. For patient ID 1542 expressing only Protein 2, spot diameters remained at 1.3 ± 0.3 mm over the 10 year observation period and for subject ID 1268 exhibiting both Protein 1 and Protein 2, the spot diameter remained constant at 1.3 ± 0.2 mm for a similar duration.

## Discussion

The identification of specific ENOX2 isoforms in sera can be indicative of the presence of cancer and also indicative of the cancer site. Malignant mesothelioma is characterized by the presence of two ENOX2 protein species of molecular weight 64 and 41 kDa, and pI 3.9 and 4.3, respectively.

All 17 patients who were diagnosed with malignant mesothelioma displayed both mesothelioma-specific protein ENOX2 isoforms. Importantly, both ENOX2 isoforms were required for a correct identification of malignant mesothelioma by using the ONCOblot test (Table 1).

Of the cancer types examined to date, the ONCOblot pattern for malignant mesothelioma most closely resembles that of bladder cancer (Table 1). However, the pI of the larger molecular weight transcript variant is sufficiently different to avoid mischaracterization (Table 1). In any case, differentiating these two cancers rarely presents a diagnostic dilemma clinically. For malignant mesothelioma two ENOX2 protein transcript variants are evident; indeed multiple ENOX2 isoforms are seen in approximately half of the different types of cancer (Table 1).

The two ENOX2 transcripts were apparent in the seven mesothelioma patients examined, 4–11 years before the clinical onset of disease (Fig. 2). This is an exciting finding and implies that production of ENOX2 proteins are an early event in carcinogenesis. To our knowledge, this is the earliest prediagnostic indicator of cancer thus far reported. The use of serum biomarkers for the early detection of cancer has been the goal of many individual researchers and research consortia, such as the Early Detection Research Network [16, 17]. While some serum biomarkers have been described, few are used in routine clinical practice [18], and most give a lead time of less than a year [19]. Biomarker utility is hampered by low levels of specificity combined with a propensity to yield false positives [20]. The only useful biomarker for mesothelioma is mesothelin which is elevated in between 15 and 40 % of individuals exposed to asbestos before diagnosis of mesothelioma [7]. Asbestos-exposed individuals represent an ideal cohort to evaluate prospective serum biomarkers for cancer detection, due to both their quantifiable exposure to a carcinogen and the well-established link to a specific cancer type, malignant mesothelioma.

The distribution of histological asbestos-related lung cancer is similar to that of lung cancers of other etiologies [21, 22]. The test has been evaluated previously for both non-small cell and small cell lung cancers (Table 1). Those cancers exhibit a single ENOX2 protein unique to lung cancer but can be distinguished by their pIs [8].

Of the subjects with benign disease, 60 % lacked ENOX2 proteins in their serum (Table 3). Both protein transcript variants were found in the serum of only one subject currently diagnosed with benign disease (subject ID 1268). For the remaining five subjects diagnosed with benign disease, only one protein transcript variant was detected. It is possible that the presence of one of the two mesothelioma-specific isoforms is an indicator of early pathological changes that predate the development of mesothelioma, as the transition from benign disease to malignant mesothelioma may be required for both transcript variants to be present. This issue would require longer follow-up to elucidate. Within the ONCOblot test, the presence of only the high molecular weight mesothelioma-specific ENOX2 transcript variant would be identified as ‘not in the database’ as no malignancy characterized to date produces a single ENOX2 transcript variant with a similar molecular weight and pI as this ENOX2 transcript variant. In contrast, the presence of only the low molecular weight transcript variant would be misidentified as a cancer of blood cell origin (Table 1).

Of note, for the three patients who were diagnosed with benign disease and examined in a time series, the detected ENOX2 spot size either remained constant or declined during the observational period. Two of these subjects (subject ID 1542 and 4288) produced only one ENOX2 transcript variant (Table 3). Although the remaining patient (subject ID 1268) produced both mesothelioma-specific transcript variants (Table 3), a steady decline of Protein 1–40 % of the initial amount detected was observed over a 9 year period. The largest spot diameter encountered in patients clinically diagnosed with mesothelioma was 6.6 mm representing a nearly tenfold increase in ENOX2 proteins in the serum compared to levels giving rise to a 2 mm diameter spot at early detection. It is possible, though not proven, that as the controls in this study have all been exposed to asbestos that these false positives may represent a pre-malignant stage of mesothelioma that has yet to become clinically meaningful. Furthermore, mesothelioma is recognized for the long latency period between asbestos exposure and malignancy, so it is possible that during this phase equilibrium is maintained between the host and the cancer. The presence of one of the mesothelioma-associated ENOX2 transcript variants may reflect this interaction. The immune system is capable of influencing the outcome of mesothelioma patients, as evidenced by the occasional finding of spontaneous mesothelioma regression accompanied by strong lymphocyte infiltration [23] and by spontaneous humoral responses [24]. This notion will be investigated further.

A test that can detect mesothelioma at an early stage might offer the prospect of early intervention as an approach to improve patient outcomes. The data from this study demonstrate that serum ENOX2 proteins characteristic of malignant mesothelioma can be detected in subjects 4–11 years before diagnosis based on clinical symptoms, and raises the possibility that the benefits of early intervention could be studied in such individuals.

## Conclusions

In a population of asbestos-exposed subjects who eventually developed malignant mesothelioma, ENOX2 transcript variants characteristic of malignant mesothelioma were present in serum 4–10 years in advance of clinical symptoms. As with all biomarker studies, these observations require validation in a larger, independent cohort of patients and should include prospective as well as retrospective sampling.

## Methods

### Case control identification

Serum samples were randomly selected from individuals who participated in an ongoing cancer prevention program [12, 13]. Samples were chosen from individuals with (1) a diagnosis of malignant mesothelioma confirmed by the Western Australian Mesothelioma Registry [25] and (2) from asbestos-exposed control subjects with benign pleural plaques either alone or with accompanying asbestosis [26]. For a sub-set of subjects, annually collected longitudinal serum samples were available. This study was approved by the Sir Charles Gairdner Hospital Human Research Ethics Committee.

### ONCOblot tissue of origin cancer detection test

Serum samples were analyzed for the presence of ENOX2 protein transcript variants by using the ONCOblot Tissue of Origin Cancer Detection Test as described [8–10]. Briefly, 25 µl of serum were separated using two-dimensional gel electrophoresis with isoelectric focusing in the first dimension to determine pI and SDS–polyacrylamide gel electrophoresis in the second dimension to determine molecular weight. Proteins were transferred to nitrocellulose and ENOX2 proteins were identified by immunoblot analysis with an ENOX2-specific recombinant antibody linked to alkaline phosphatase using a colorimetric substrate for detection. Blots were scanned and the pI and molecular weight of each ENOX2 transcript variant present was calculated by comparison to molecular weight standards and to two internal reference proteins (serotransferrin, ca. 82 kDa, pI 6.8, and α-fetuin, ca. 53 kDa, pI 4.1). The two reference serum proteins, serotransferrin and α-fetuin are detected on western blots because they both share a similar five amino acid sequence within the antibody combining site shared by all ENOX2 protein transcript variants [10]. Transferrin or α-fetuin antibodies were not added nor is the reaction unspecific. Each transcript variant of ENOX2 migrates to a specific location defined by the two reference proteins and has a specific location (molecular weight and pI) on the blot that correlates to the known tissue of origin determined from banked sera samples collected from individuals with a clinically confirmed diagnosis of cancer (Table 1).

The protein chemistry differences that underlie the molecular weight and pI differences that distinguish tissue-specific ENOX2 transcript variants result from alternative splicing of the ENOX2 mRNA [27]. Each of the protein transcript variants share a common exon 5 which contains the ENOX2-specific antibody-combining site including the amino acid sequence EEMTE. The overall sensitivity of the test is estimated to be >95 %.

The different tissues of origin provide for non-overlapping patterns of ENOX2 protein transcript variants each with a characteristic number of protein transcript variants, molecular weights and pIs. The majority of the tissues of origin, including small cell and non-small cell lung cancer, are represented by a single transcript variant. Several tissues of origin, including mesothelioma, ovarian, hepatocellular, uterine and six others are represented by two transcript variants. Three cancer tissues of origin, stomach, colon and kidney, have three [8].

### Quantitation of ENOX2 based on spot diameter

To estimate relative amounts of ENOX2 in sera, data from patients were compared to a standard curve of known amounts of a functional, 46 kDa form of recombinant human ENOX2 generated in *E coli*. The log of the spot diameter and the log of the mass of ENOX2 detected by immunoblot after 2-D separation correlated linearly, r^2^ = 0.95 (Fig. 3). The complete amino acid sequence of a 72 kDa form of ENOX2 is available from GenBank under accession no. AF207881. The limit of detection of the ONCOblot assay is approximately 100 femtomoles of an ENOX2 protein [11].

## References

[1] Robinson BW, Musk AW, Lake RA (2005). Malignant mesothelioma. Lancet.

[2] Robinson BW, Lake RA (2005). Advances in malignant mesothelioma. N Engl J Med.

[3] Antman K, Pass H, Schiff P, DeVita VT, Heliman S, Rosenberg S (2001). Benign and malignant mesothelioma. Cancer: principles and practice of oncology.

[4] Vongelzang NJ, Rusthoven JJ, Symanowski J, Denham C, Kaukel E, Ruffie P (2003). Phase III study of pemetrexed in combination with cisplatin versus cisplation alone in patients with malignant pleural mesothelioma. J Clin Oncol.

[5] Flores RM, Pass HI, Seshan VE, Dycoco J, Zakowski M, Carbone M, et al. Extrapleural pneumonectomy versus pleurectomy/decortication in the surgical management of malignant pleural mesothelioma: results in 663 patients. J Thorac Cardiovasc Surg 2008;135:620–6, 626 e1–3. doi: 10,1016/j.jtcvs.2007.10.054. 10.1016/j.jtcvs.2007.10.054 18329481

[6] Sugarbaker DJ, Flores RM, Jaklitsch MT, Richards WG, Strauss GM, Corson JM, et al. Resection margins, extrapleural nodal status, and cell type determine postoperative long-term survival in trimodality therapy of malignant pleural mesothelioma: results in 183 patients. J Thorac Cardiovase Surg 1999;117:54–63. discussion 63–5. 10.1016/s0022-5223(99)70469-1 9869758

[7] Creaney J, Olsen NJ, Brims F, Dick IM, Musk AW, de Klerk NH (2010). Serum mesothelin for early detection of asbestos-induced cancer malignant mesothelioma. Cancer Epidermiol Biomarkers Prev.

[8] Hostetler B, Weston N, Kim C, Morré DM, Morré DJ (2009). Cancer site-specific isoforms of ENOX2 (tNOX), a cancer-specific cell surface oxidase. Clin Proteomics.

[9] Morré DJ, Morré DM. Early detection: an opportunity for cancer prevention through early interventions. In: Georgakilas AG, editor. Cancer prevention. Rijeka: In Tech; 2012. pp. 389–402.

[10] Morré DJ, Morré DM (2013). ECTO-NOX Proteins.

[11] Hanau C, Morré DJ (2014). Morré DM. Cancer prevention trial of a synergistic mixture of green tea concentrate plus *Capsicum* (Capsol-T) in a random population of subjects ages 40-84. Clin Proteomics.

[12] Musk AW, de Klerk NH, Ambrosini GL, Eccles JL, Hansen J, Olsen NJ (1998). Vitamin A and cancer prevention I: observations in workers previously exposed to asbestos at Wittenoom, Western Australia. Int J Cancer.

[13] de Klerk NH, Musk AW, Ambrosini GL, Eccles JL, Hansen J, Olsen N (1998). Vitamin A and cancer prevention II: comparison of the effects of retinol and beta-carotene. Int J Cancer.

[14] Robinson BW, Creaney J, Lake R, Nowak A, Musk AW, de Klerk N (2003). Mesothelin-family proteins and diagnosis of mesothelioma. Lancet.

[15] Creaney J, van Bruggen I, Hof M, Segal A, Musk AW, de Klerk N (2007). Combined CA125 and mesothelin levels for the diagnosis of malignant mesothelioma. Chest.

[16] Srivastava S, Gopal-Srivastava R (2002). Biomarkers in cancer screening: a public health perspective. J Nutr.

[17] Greenwald P (2007). A favorable view: progress in cancer prevention and screening. Recent Results Cancer Res.

[18] Neal DE, Donovan JL, Martin RM, Hamdy FC (2009). Screening for prostate cancer remains controversial. Lancet.

[19] Shih L-M, Sokoll LJ, Diamandis EP, Fritsche HA, Lilia H, Chan DW, Schwartz MK (2002). Chan DW. Ovarian cancer, tumor markers.

[20] Moss EL, Hollingworth J, Reynolds TM (2005). The role of CA125 in clinical practice. J Clin Pathol.

[21] Roggli VL, Sanders IL (2000). Asbestos content of lung tissue and carcinoma of the lung: A clinicopathologic condition and mineral fiber analysis of 234 cases. Ann Occup Hyg.

[22] Lee BW, Wain JC, Kelsey KT, Wiencke JK, Christiani DC (1998). Association of cigarette smoke and asbestosis exposure with location and histology of lung cancer. Am J Respit Crit Care Med.

[23] Robinson BWS, Robinson C, Lake RA (2001). Localized spontaneous regression in mesothelioma—possible immunological mechanism. Lung Cancer.

[24] Ho M, Hassan R, Zhang J, Wang QC, Onda M, Bera T, Pastan I (2005). Humoral immune response to mesothelin in mesothelioma and ovarian cancer patients. Clin Cancer Res.

[25] Threitall T, Thompson J, Olsen N. Cancer in Western Australia: incidence and mortality 2003 and mesothelioma 1960-2003. Department of health, statistical series number 74. Perth: 2005.

[26] Musk AW, de Klerk NH, Reici A, Ambrosini GL, Fritschi L, Olsen NJ (2008). Mortality of former crocidolite (blue asbestos) miners and millers at Wittenoom. Occup Enviorn Med.

[27] Tang X, Tian Z, Chueh P-J, Chen S, Morré DM, Morré DJ (2007). Alternative splicing as the basis for specific localization of tNOX, a unique hydroquinone (NADH) oxidase to the cancer cell surface. Biochemistry.

