# Erratum to: ENOX2-based early detection (ONCOblot) of asbestos-induced malignant mesothelioma 4–10 years in advance of clinical symptoms

**DOI:** 10.1186/s12014-016-9105-1

**Published:** 2016-02-22

**Authors:** D. James Morré, Brandon Hostetler, David J. Taggart, Dorothy M. Morré, A. W. Musk, Bruce W. S. Robinson, Jenette Creaney

**Affiliations:** MorNuCo, Inc, Purdue University Research Park, 1201B Cumberland Avenue, West Lafayette, IN USA; National Centre for Asbestos Related Disease, University of Western Australia, Perth, Australia; School of Medicine and Pharmacology, University of Western Australia, Perth, Australia; Department of Respiratory Medicine, Sir Charles Gairdner Hospital, Nedlands, Australia; School of Population Health, University of Western Australia, Perth, Australia

## Erratum to: Clin Proteom (2016) 13:2 DOI 10.1186/s12014-016-9103-3

It has come to the publisher’s attention that the original version of this article [1] unfortunately contained an unnecessarily complicated version of the data in Table 1. This Erratum replaces Table 1 of the original article with the table below.

**Table 1:** Range of molecular weights and isoelectric points (99th percentile) of ENOX2 transcript variants for 16 common cancer types compared with mesothelioma [Unpublished data from the ONCOblot Tissue of Origin Database, MorNuCo, Inc]

Cancer	N	Acceptable ranges					
		Protein 1		Protein 2		Protein 3	
		MW (kDa)	pI (pH)	MW (kDa)	pI (pH)	MW (kDa)	pI (pH)
Bladder	25	63–66	4.2–5.6	42–48	4.1–4.8		
Blood cell^a^	82	34–47	3.5–4.5				
Breast	538	64–69	4.2–4.9				
Cervical	37	90–100	4.2–5.4				
Colorectal^b^	90	80–96	4.4–5.4	50–65	4.2–5.3	33–46	3.8–5.2
Endometrial (uterine)	43	67–71	4.2–5.1	41–48	3.7–5.4		
Gastric	10	120–188	4.7–5.5	50–62	4.5–5.6	45–53	2.4–3.6
Hepatocellular	19	58–70	4.5–5.0	34–40	4.1–5.2		
Lung	200	52–56	4.1–5.3				
Melanoma	39	37–41	4.6–5.3				
Mesothelioma^c^	18	60–68	3.8–4.1	38–44	3.8–4.6		
Ovarian	98	72–90	3.7–5.0	37–47	3.7–5.0		
Pancreatic	61	49–51	3.9–5.4				
Prostate	178	71–88	5.1–6.5				
Kidney (renal cell)	21	69–73	4.7–5.4	54–61	4.1–5.2	38–43	3.7–4.3
Sarcoma	20	50–55	5.2–5.6	37–45	4.3–4.9		
Squamous cell	41	57–68	5.0–5.4				
Total	1520						

^a^Includes leukemia, lymphoma and myeloma

^b^In approximately 50 % of subjects, the larger transcript variant is absent

^c^Present study
